# FACTORS ASSOCIATED WITH HAND HYGIENE COMPLIANCE OF NURSING STAFF IN LONG-TERM CARE FACILITIES

**DOI:** 10.1093/geroni/igae098.2797

**Published:** 2024-12-31

**Authors:** EunKyo Kim, Eunhee Cho, Gwang Suk Kim, Kyung Hee Lee

**Affiliations:** Yonsei University, Seoul, Republic of Korea; Yonsei University, Seoul, Republic of Korea; Yonsei University, Seoul, Republic of Korea; Yonsei University, Seoul, Republic of Korea

## Abstract

This study was performed to identify hand hygiene compliance factors of registered nurses (RN) and nurse aids (NA) in long-term care facilities. This cross-sectional study surveyed 78 RNs and 50 NAs who worked at long-term care facilities. Data were collected from October 1st to December 30th, 2020. The hand hygiene knowledge, perception, and infrastructure were measured by translated versions of the World Health Organization(WHO)’s hand hygiene knowledge, perception, and infrastructure tool(2009). Hand hygiene compliance was calculated as ‘number of conducted WHO’s hand hygiene at five moments’ out of ‘number of chances to conduct WHO’s hand hygiene at five moments’ multiplied by 100, which participants wrote. Data were analyzed using Spearman’s rank correlation coefficient and multiple linear regression. The hand hygiene compliance rate was 78.85%. The mean score of hand hygiene knowledge was 13.55±2.21 out of 25 points, the perception mean score was 73.41±11.65 out of 96 points, and the infrastructure mean score was 32.42±5.18 out of 40 points. Hand hygiene compliance positively correlated with the perception (r=0.177, p=0.045) and the infrastructure(r=0.194, p=0.028). Factors influencing hand hygiene compliance were the infrastructure (β=0.223, p=0.042) and working in a specialized unit (β=0.221, p=0.013) for residents who need nursing treatments (e.g., indwelling catheter exchange and wound management) and with mandating minimum registered nurse staffing level. Hand hygiene compliance was affected by infrastructure and working units with different nursing staffing levels. The results suggest expanding the infrastructure and ensuring adequate nursing staffing in long-term care facilities to improve hand hygiene compliance.

